# Instruction of Immunometabolism by Adipose Tissue: Implications for Cancer Progression

**DOI:** 10.3390/cancers13133327

**Published:** 2021-07-02

**Authors:** Remya Raja, Christopher Wu, Francesca Limbeck, Kristina Butler, Abhinav P. Acharya, Marion Curtis

**Affiliations:** 1 Department of Immunology, Mayo Clinic, Scottsdale, AZ 85259, USA; raja.remya@mayo.edu (R.R.); wu.christopher@mayo.edu (C.W.); chessie.dancer@gmail.com (F.L.); 2 Division of Gynecologic Surgery, Mayo Clinic, Phoenix, AZ 85054, USA; Butler.Kristina@mayo.edu; 3 Department of Chemical Engineering, School for the Engineering of Matter, Transport and Energy, Arizona State University, Tempe, AZ 85281, USA; abhi.acharya@asu.edu; 4 Department of Cancer Biology, Mayo Clinic, Scottsdale, AZ 85259, USA; 5 College of Medicine and Science, Mayo Clinic, Scottsdale, AZ 85259, USA

**Keywords:** immunometabolism, adipose tissue, adipocyte, immune cell, cancer, metastasis

## Abstract

**Simple Summary:**

Metabolism is the process by which living organisms and cells generate energy to sustain life. At the organismal level, metabolic homeostasis is a tightly controlled balance between energy consumption and energy expenditure. Many studies have shown that disruption of this homeostasis leads to an inflammatory phenotype within adipose tissue. The aim of this review is to provide an overview of the dynamic metabolic interplay within adipose tissue and its implications for cancer progression and metastasis.

**Abstract:**

Disruption of metabolic homeostasis at the organismal level can cause metabolic syndrome associated with obesity. The role of adipose tissue in cancer has been investigated over the last several decades with many studies implicating obesity as a risk factor for the development of cancer. Adipose tissue contains a diverse array of immune cell populations that promote metabolic homeostasis through a tightly controlled balance of pro- and anti-inflammatory signals. During obesity, pro-inflammatory cell types infiltrate and expand within the adipose tissue, exacerbating metabolic dysfunction. Some studies have now shown that the intracellular metabolism of immune cells is also deregulated by the lipid-rich environment in obesity. What is not fully understood, is how this may influence cancer progression, metastasis, and anti-tumor immunity. This review seeks to highlight our current understanding of the effect of adipose tissue on immune cell function and discuss how recent results offer new insight into the role that adipose tissue plays in cancer progression and anti-tumor immunity.

## 1. Introduction

Recently, metabolism, both at the organismal and cellular levels, has been found to play a pivotal role in many disease states such as obesity and cancer. Adipose tissue is a critical regulator of organismal metabolism and deregulation of adipose tissue homeostasis can lead to metabolic dysfunction culminating in obesity [1]. Co-morbidities associated with obesity such as type 2 diabetes, heart disease, and cancer, put a large burden on health care systems and drastically reduce the quality and length of life [2]. Several cancers (i.e., breast, ovarian, colon, pancreatic) either develop in or metastasize to adipose-rich tissues. A better understanding of how adipose tissue may regulate the metabolism of immune cells during cancer progression has the potential to greatly impact cancer therapy.

In this review, we seek to provide an overview of the current understanding of how adipose tissue influences the metabolism and function of immune cells with a primary focus on visceral deposits of white adipose tissue. Most of the current studies investigating the relationship between adipose tissue and immune cells are centered on the study of the obese versus lean state. This review seeks to summarize these findings and to discuss how these studies provide insight into the role of adipose-immune crosstalk in cancer progression, metastasis, and anti-tumor immunity.

## 2. Adipose Tissue in Cancer

Adipose tissue can be broadly grouped into three main types—white, brown, and beige, which have different anatomic distributions and homeostatic functions [3]. White adipose tissue (WAT) is the primary site for energy storage and because of its additional role as an endocrine organ, WAT has been implicated as the main player in the development of obesity and metabolic disorders. WAT can be further sub-divided into either subcutaneous (SAT) or visceral (VAT) adipose tissue. In humans, SAT is thought to serve as a barrier to mechanical injury while VAT protects visceral organs and is the main storage site for triglycerides. Vertebrates utilize a specialized cell type called an adipocyte to store energy in the form of triglycerides that are hydrolyzed to glycerol and fatty acids that can then be transported throughout the body.

Adipocytes produce hormones, known as adipokines, which have significant autocrine, paracrine, and endocrine implications especially in the context of cancer. Adipokines, such as leptin, adiponectin, visfatin, and omentin, play important roles in the modulation of energy expenditure, but also possess crucial immunomodulatory abilities [4]. Leptin is the main negative regulator of food intake. Disruption of the leptin signaling axis in animal models leads to obesity and leptin resistance is a hallmark of metabolic disorder in humans [5,6]. Leptin was also found to be a potent stimulator of immune function by inducing proliferation of human T cells and reversing starvation induced immunosuppression [7]. Adiponectin also plays significant roles in metabolic homeostasis and has important anti-inflammatory properties [8,9,10]. The decrease of adiponectin, which is commonly associated with obesity, results in increased secretion of pro-inflammatory cytokines including TNF-α [11]. In most obesity-associated cancer types, adiponectin inhibits tumor growth in vitro and is associated with improved survival [11]. Due to their ability to modulate both cancer signaling and immune function, adipokines and their receptors have the potential to be important therapeutic targets in cancer.

### 2.1. Obesity and Cancer Risk

**Figure 1:**
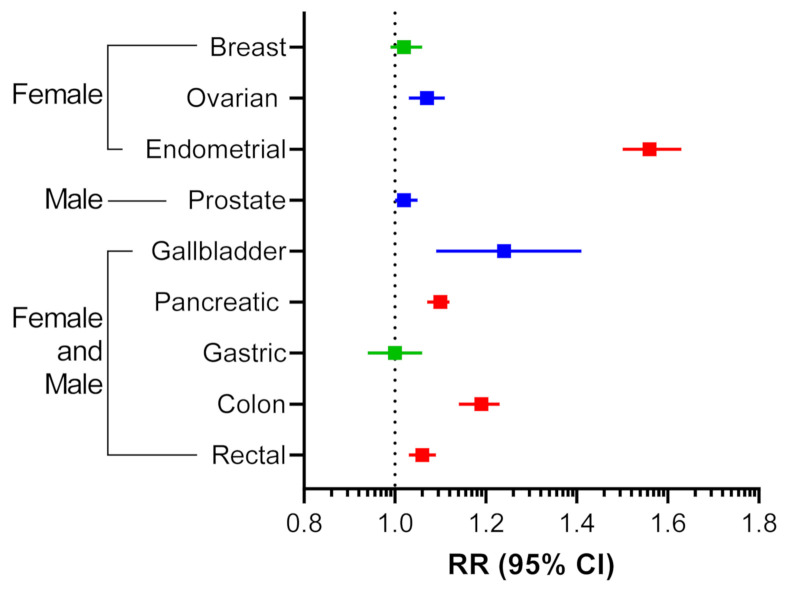
Associations between BMI increase and cancer risk. Relative risk (RR) association as defined by random effects is shown with 95% confidence interval (CI). Red, *p* < 0.001; Blue, 0.001 ≤ *p* < 0.05; Green, *p* ≥ 0.050. Adapted from Table 4 of [15].

With the increasing incidence of obesity in the United States and globally, there has been growing epidemiological evidence identifying obesity as a risk factor for cancer development, negative prognosis, and resistance to delivery of systemic therapies [12,13]. Reanalysis of the most extensive meta-analysis to date, looking at 221 datasets between 1966 to 2007, reported strong associations between increases in BMI and cancer risk [14,15]. Endometrial cancer was found to have the highest association with obesity (Figure 1). Gallbladder, ovarian, and colon cancers also had strong associations with obesity while breast and gastric cancers were found to be non-significant (Figure 1). Overall, it is clear that there is a cohort of cancer types that is highly associated with obesity. Therefore, elucidating the role that adipose tissue plays in cancer progression will be critical to the development of effective cancer therapies.

### 2.2. The Role of Visceral Adipose Tissue in Cancer

As the primary organ involved in obesity, the role of adipose tissue in cancer growth and metastasis has garnered increased interest over the last decade [13,16]. The omentum is a 20 × 20 × 3 cm pad of VAT, which is a common site of metastasis in many peritoneally disseminating cancers including ovarian, gastric, colon, and pancreatic cancers. In the non-disease state, this organ is primarily composed of adipocytes covered by a single layer of mesothelial cells with fibroblasts below a basement membrane. Following metastasis, the omentum transforms into a solid tumor with fibroblast-rich stroma, largely devoid of adipocytes. The omentum is known to play an active role in immunity [17,18] first recognized in the 1800s, by expanding leukocyte centers (milky spots) and by physically covering foreign agents during infection [19]. Omental metastasis is mainly attributed to released chemoattractants and lipid-rich stores found within adipocytes that serve as a source of energy for metastatic tumor cells [20,21]. However, a recent study identified creatine synthesis within adipocytes as a central mediator of obesity-induced cancer growth in a syngeneic mouse model of breast cancer [22]. Therapies targeting lipid transport, lipid utilization, or creatine biosynthesis may prove to be beneficial for cancer patients, especially patients whose cancer is associated with obesity.

## 3. Adaptive Immune Cell Metabolism and Adipose Tissue

**Figure 2:**
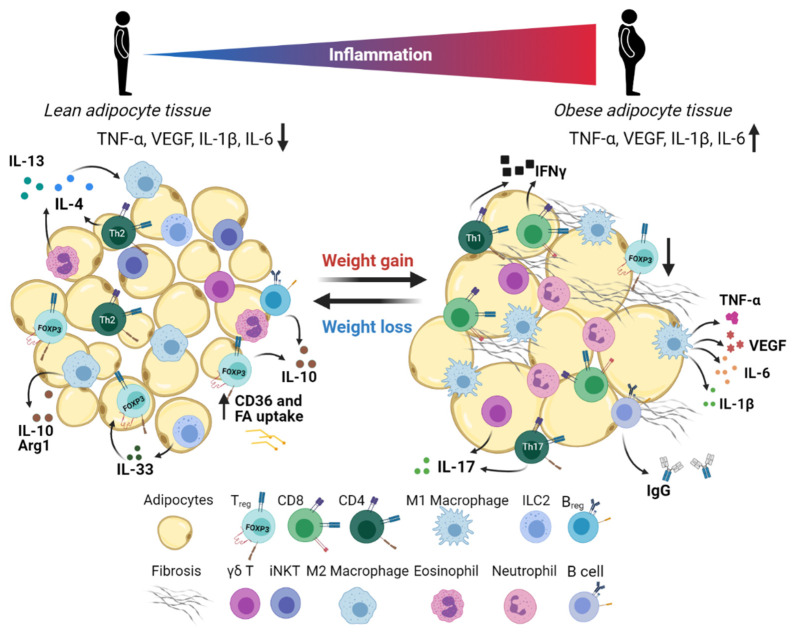
Snapshot of omental microenvironment during lean vs. obese conditions. WAT plays a crucial role in energy homeostasis. White adipocytes act as the primary site for storage of triglycerides, which then serve as an energy reservoir during conditions of high energy consumption or fasting. Healthy adipose tissue is enriched in regulatory T cells (T_reg_), M2 macrophages, invariant natural killer T (iNKT) cells, γδ T cells, type 2 innate lymphoid cells (ILC2s), eosinophils, and regulatory B cells (B_reg_). These immune cells secrete various anti-inflammatory cytokines such as IL-10, IL-33, and IL-4 that help to bring inflammation under control. Under obese conditions, WAT becomes severely dysfunctional. Hypertrophic adipocytes and adipose tissue fibrosis are characteristic features of dysfunctional WAT. Further, obesity induces local inflammation with M1 macrophages playing a leading role via secretion of pro-inflammatory cytokines including TNF-α and IL-6. The pro-inflammatory milieu perturbs the immune cell composition with an increased presence of CD8 T cells, type 1 T helper (Th1) cells, and neutrophils. Accumulating M1 macrophages secretes VEGF, resulting in aberrant vascularization in the WAT. Failure to restore normal WAT homeostasis leads to systemic low-grade inflammation and metabolic syndromes.

Although adipose tissue is primarily composed of adipocytes, a diverse repertoire of stromal cells also takes up residence in WAT. These cells are collectively referred to as the stromal vascular fraction (SVF), which includes the preadipocytes, mesothelial cells, endothelial cells, fibroblasts, stem cells, and immune cells (Figure 2). Immune cells have been known to infiltrate WAT for a long time, but the initial link between adipose tissue and inflammation was first discovered by Hotamisligil et al. in 1993 when the group identified increased TNF-α levels in adipose tissue of obese mice [23]. Disruption of the homeostatic balance between pro- and anti-inflammatory cellular mediators in adipose tissue is a basis for the development of metabolic disorders and may have important implications for cancer metastasis.

Adaptive immunity has been shown to play an indispensable role in organismal immunometabolism. Here, we seek to highlight key studies that contribute to our understanding of the role of adaptive immune cells in organismal and cellular immunometabolism along with their role in anti-tumor immunity.

### 3.1. CD4 T Helper Cells

CD4 T helper cells are a heterogeneous group of cells that play a critical role in the adaptive immune response by recruiting and activating other immune cells. Based on their function, transcription factor expression, and the cytokines they produce, CD4 T helper cells can be sub-divided into three major lineages: Th1, Th2, and Th17 cells. While the majority of immunotherapies have focused on harnessing CD8 T cells, recent studies have illuminated the important role of CD4 T cells in anti-tumor immunity [24]. Pro-inflammatory cytokine production in obese adipose tissue has historically been attributed to the innate immune system, specifically macrophages [25], which will be discussed later on. However, many studies have now demonstrated that the adaptive immune response also plays a role in stimulating adipose tissue inflammation. Th1 cells are a member of the adaptive immune system that promotes inflammation in adipose tissue through the secretion of IFNγ and the activation of macrophages [26]. Th1 cells were found to infiltrate adipose tissue prior to the arrival of macrophages in a mouse model of obesity [27]. Mice lacking conventional T cells were partially protected against obesity-induced insulin resistance and had reduced macrophage infiltration in their adipose tissue [28]. These findings highlight the significant role of Th1 cells in modulating the pro-inflammatory response in adipose tissue during obesity.

Compared to other immune cell types, there have been relatively few studies that have investigated the role of Th2 cells in adipose tissue inflammation. Th2 cells are considered an anti-inflammatory cell type that secretes IL-4, IL-5, IL-10, and IL-13, which counterbalances the Th1 immune response [29]. One of the functions of Th2 cells is to induce the differentiation of macrophages into an anti-inflammatory subtype (M2) that secretes IL-10 [30]. Winer et al. found that nearly three times more Th1 cells accumulated in the VAT of mice fed a high-fat diet compared to those fed a normal diet, whereas anti-inflammatory Th2 and T_reg_ cells did not change in abundance [31]. These findings were confirmed in human samples where T cell profiling in VAT from obese patients revealed that the Th2 frequency and IL-10 expression in VAT inversely correlated with insulin resistance [32]. Taken together, these studies demonstrate that Th2 cytokines in adipose tissue are protective against metabolic dysfunction.

IL-17 is a pro-inflammatory cytokine that acts on a variety of stromal cells to stimulate the production of diverse pro-inflammatory cytokines during an immune response [33]. Th17 cells are a subset of CD4 effector T cells that are so named due to their production of IL-17A. Plasma levels of IL-17 are higher in obese patients [34] and Th17 cells have been reported to be increased 3- to 10-fold in the SAT from obese patients with insulin resistance compared to lean patients [35]. However, IL-17 expression is not limited to Th17 cells. CD8 T cells, γδ T cells, NK cells, and neutrophils are also sources of IL-17 [33] and studies in obese mice have indicated that the γδ T cells are a primary source of IL-17 in adipose tissue [36,37].

γδ T cells are an innate T cell type that does not recognize antigen in the context of MHC. Given the ability of adipose tissue-resident γδ T cells to produce large amounts of IL-17, we will include them here along with the discussion of Th17 cells. High levels of Th17 and γδ T cells correlate with increased obesity in mice and humans, which would suggest that IL-17 plays a pro-inflammatory role in obesity. However, evidence from mouse models and in vitro experiments indicates that IL-17′s role in obesity is more complicated. Mice deficient in IL-17 [36] or the receptor for IL-17 (IL-17RA) [38] that were fed a high-fat diet had significantly greater weight gain but were protected from dysregulation of glucose metabolism [36]. Correspondingly, mice deficient in γδ T cells that were fed a high-fat diet had significant reductions in inflammation and insulin resistance, while having no effect on obesity [39].

IL-17 displays disparate biological effects on anti-tumor immunity. The presence of intertumoral γδ T cells is the most significant predictor of favorable cancer outcome in a pan-cancer analysis as compared to all other leukocyte populations [40]. Many studies show that Th17 cells can be either pro-tumorigenic or anti-tumorigenic, indicating that the role of IL-17 in anti-tumor immunity is highly context and cancer-type dependent [41]. Notably in ovarian cancer, which primarily metastasizes to the omentum, Th17 cell presence significantly correlated with better prognosis [42]. Alternatively, in a study using a mouse model of spontaneous breast cancer metastasis, IL-17-expressing γδ T cells promoted metastasis through an IL-17/neutrophil axis but did not affect primary tumor growth [43]. Therefore, current studies point to the important role of IL-17-producing T cells in the regulation of glucose homeostasis and during cancer progression. Further research is needed to elucidate the complex role of IL-17 in specific disease settings.

### 3.2. Regulatory T (T_reg_) Cells

One of the best-studied adaptive immune cell types in adipose tissue is a unique subset of CD4, Foxp3-positive regulatory T (T_reg_) cells. T_reg_ cells play a critical role in the maintenance of immune homeostasis by suppressing auto-immune T cell reactions in the periphery. In cancer, T_reg_ cell suppression of anti-tumor immunity is a major barrier to productive anti-tumor immune responses and response to immunotherapy [44]. VAT T_reg_ cells have been shown to be central players in maintaining adipose tissue homeostasis through their anti-inflammatory properties [45]. T_reg_ cells in VAT from lean mice are enriched compared to lymphoid organs and are significantly reduced with diet-induced obesity. Interestingly, VAT T_reg_ cells have a unique transcriptomic profile distinct from T_reg_ cells residing in lymphoid tissue with increased transcript levels for genes involved in lipid metabolism (*CD36*, *DGAT1*, *LDLR*) [45]. The accumulation and phenotype of T_reg_ cells in VAT of lean mice is driven by the expression of peroxisome proliferator-activated receptor γ (PPARγ) [46]. PPARs (α, β, δ, γ) are nuclear receptors that are activated by lipid-derived substrates and are the central mediators of energy homeostasis. PPARγ regulates many metabolic processes through activation of transcription including fatty acid uptake, glucose uptake, and lipogenesis in adipose tissue [47]. In response to a PPARγ agonist, naïve CD4 T cells drastically upregulated many genes involved in lipid metabolism [46].

In cancer, an increased prevalence of circulating and intra-tumoral T_reg_ cells has been observed [48,49]. In addition, the high frequency of Foxp3-positive T_reg_ cells has been shown to be associated with an adverse outcome in multiple cancer types [50,51,52]. Similar to VAT T_reg_ cells, tumor-derived T_reg_ cells also upregulate lipid metabolism and CD36 through a PPARβ-dependent mechanism [53]. CD36 expression sustained survival of intertumoral T_reg_ cells by promoting mitochondrial fitness. Genetic deletion of CD36 in T_reg_ cells led to decreased T_reg_ cell infiltration into tumors and greater anti-tumor immunity [53]. Modulation of T_reg_ cell populations through inhibition of CD36 or activation of lipid metabolism via PPARγ agonists may be viable strategies to limit T_reg_ cell accumulation within tumors.

### 3.3. CD8 T Cells

CD8 T cells are a subset of T lymphocytes known for their pro-inflammatory and cytotoxic abilities. CD8 T cells are the main mediators of the cytotoxic anti-tumor immune response and their presence within tumors correlates with enhanced survival [54,55] and improved response to immunotherapy [56,57]. Many studies have shown infiltration of CD8 T cells in adipose tissue under obese conditions in humans as well as in mice [32,58,59]. IFNγ derived from CD8 and Th1 cells has been shown to induce adipocyte dysfunction, causing impaired glucose uptake, impaired lipid storage, and reduced adipocyte differentiation [60]. Furthermore, it was demonstrated that genetic depletion of CD8 T cells reduced macrophage infiltration and adipose tissue inflammation, which suppressed insulin resistance [61]. Expression of perforin and granzymes, which are effector molecules produced by activated CD8 T cells, are also increased in VAT of high fat diet-fed mice [62]. Therefore, CD8 T cells play a mostly pathogenic role in obesity.

**Figure 3:**
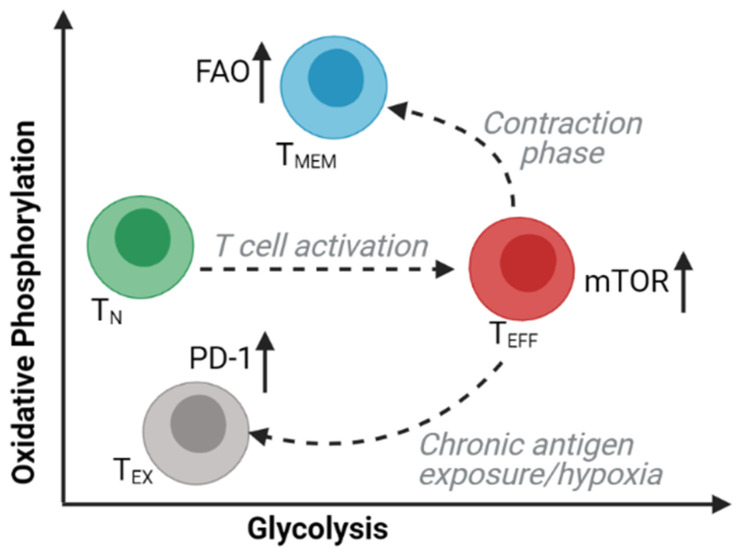
Metabolism as a driver of T cell function. During its life cycle, T cells rely on specific metabolic pathways to satisfy the energy demand during differentiation and activation. Mature naive T cells (T_N_) that exit from the thymus are more reliant on oxidative phosphorylation (OXPHOS). Upon encounter with antigen, T cells undergo proliferation and preferentially engage aerobic glycolysis through the action of mTOR to sustain the requirements of proliferation and effector function. The antigen-specific activated effector T cell (T_EFF_) resulting from clonal expansion is metabolically distinct as it engages both aerobic glycolysis and OXPHOS pathway. Differentiation of effector T cells to memory T cells (T_MEM_), switches the metabolic phenotype back to OXPHOS driven by fatty acid oxidation (FAO). However, upon secondary antigen exposure, T_MEM_ cells rely on both OXPHOS and glycolysis to facilitate recall response. Chronic antigenic stimulation and exposure to inhibitory ligands can lead to a decline in T cell function, referred to as T cell exhaustion (T_EX_), which sees a marked reduction in both OXPHOS and glycolic activity.

Memory T (T_MEM_) cells are long-lived subsets of CD8 and CD4 T cells that are responsible for immunological memory following an acute immune response. The presence of central memory and stem-cell memory phenotypes correlates with successful outcomes from adoptive cell immunotherapy in mice [63] and humans [64]. Studies of intracellular bioenergetic profiles have revealed the extensive metabolic changes that occur in T cell subsets during differentiation and activation (Figure 3). T_MEM_ cells adopt a metabolic profile that is distinct from other T cell subsets and is characterized by an increased dependency on oxidative phosphorylation (OXPHOS) and a decreased reliance on glycolysis [65,66,67]. Tissue-resident memory (T_RM_) T cells are T_MEM_ cells that persist within tissues. Dissimilar to their central memory T (T_CM_) cell counterparts, T_RM_ cell persistence has been shown to require molecules that facilitate exogenous uptake, intracellular transport, and mitochondrial metabolism of lipids, including FA-binding proteins 4 and 5 (FABP4 and FABP5) [68]. Han et al. reported that approximately half of effector CD8 and CD4 T cells within the WAT may be tissue-resident memory T cells, with the remaining cells expressing an effector memory phenotype. T_RM_ cells were enriched within WAT post mucosal infections and possess an increased proliferative and metabolic capacity as well as an enhanced effector potential for subsequent infections [69].

Within tumors, the anti-tumor function of CD8 T cells is frequently compromised by the acquisition of a dysfunctional phenotype known as T cell exhaustion. T cell exhaustion is characterized by inadequate effector function, reduced metabolic potential, and increased expression of inhibitory receptors driven by distinct epigenetic and genetic programs [67,70,71,72,73]. The exhausted phenotype can be at least partially reversed by blocking the inhibitory receptor PD-1 [74]. However, nutrient and oxygen availability within the tumor microenvironment also play significant roles in modulating T cell exhaustion. Competition between T cells and cancer cells for glucose within the tumor microenvironment restricts the glycolytic capacity and IFNγ production of T cells, allowing for tumor progression [75]. Hypoxia in combination with chronic antigen stimulation is also a driver of T cell exhaustion through repression of mitochondrial function [76]. While little is currently known regarding the role of adipose tissue in T cell exhaustion, fatty acids [77], oxidized lipids [78], and cholesterol [79] were found to impede T cell effector function and anti-tumor immunity.

It is not known why CD8 T cells are able to retain their function under high lipid conditions in obesity but are compromised by lipids within the tumor microenvironment. However, this may have to do with metabolic reprogramming of the T cells or may be dependent on the differentiation state of the T cells. Identification of the factors that facilitate lipid-mediated T cell dysfunction will have important implications for the development of immunotherapies. Notably, activation of fatty-acid oxidation using a PPARα agonist was shown to reverse T cell exhaustion and synergize with a PD-1 antibody to inhibit melanoma growth [80]. Taken together, these studies highlight the potential role that adipose tissue may play in regulating the metabolic state of T cells, which may have implications in the anti-tumor immune response. The metabolic phenotype of T cells within the omentum during cancer metastasis remains largely unknown.

### 3.4. B Cells

B cells are a critical part of the adaptive immune response that mediates the antibody response to infection and cancer. B cells have been identified within all adipose tissue depots and studies have shown that B cells contribute to insulin resistance by presenting antigens to T cells, secreting inflammatory cytokines, and producing pathogenic antibodies (reviewed in [81]). B cells accumulate within VAT of mice on a high-fat diet at an early timepoint corresponding with an increase in T cells and macrophages [82]. Mice deficient in mature B cells that were fed a high-fat diet displayed reduced inflammatory T cells within VAT and improved insulin sensitivity [82,83]. However, like T cells, many subsets of B cells exist. Nishimura et al. identified a subpopulation of regulatory B cells within murine and human WAT that constitutively produce the anti-inflammatory cytokine, IL-10. The study further showed that B cell-specific deletion of IL-10 results in increased infiltration of inflammatory macrophages and CD8 T cells into adipose tissue [84]. It is currently unknown what role adipose tissue-resident B cells play in tumor progression. A detailed understanding of their impact on cancer development would be critical in improving anti-tumor immune responses.

## 4. Innate Immune Cell Metabolism and Adipose Tissue

Healthy adipose tissue contains a large assortment of innate immune cells. The frequency of these innate cells changes dramatically as the adipose tissue expands during obesity and leads to increased numbers of both anti- and pro-inflammatory cells within the tissue. Since innate immune cells are the first responders, this effect can be measured in the innate immune cell frequencies. This section will describe the role of different innate immune cells in generating immune responses within adipose tissue and their implications for cancer metastasis.

### 4.1. Macrophages

**Figure 4:**
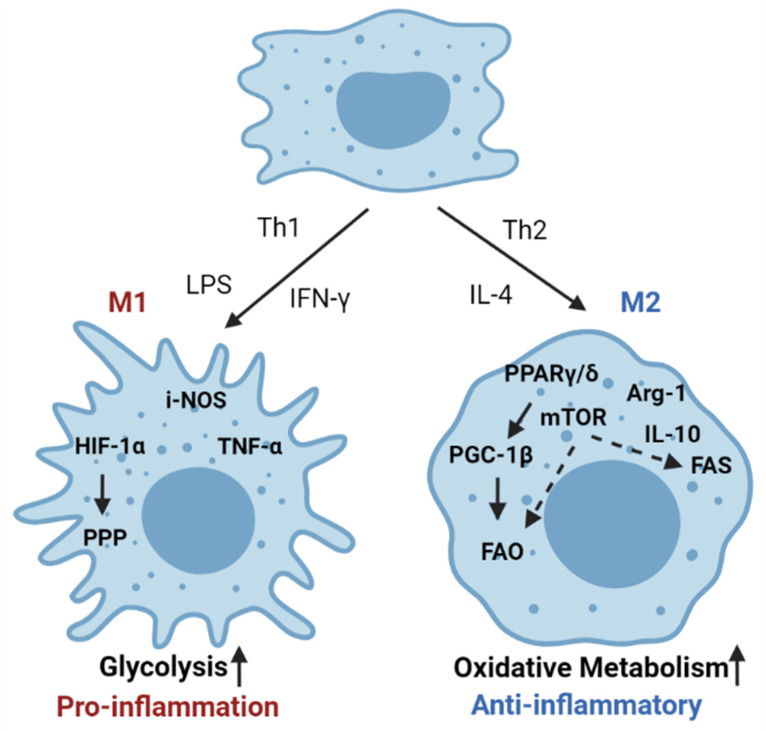
Metabolic pathways in macrophage activation. Macrophages are the key players involved in adipose tissue homeostasis. Under obese conditions, there is an influx of pro-inflammatory macrophages (M1). M1 macrophages have increased HIF1α levels that result in increased glycolysis and engagement of the pentose phosphate pathway (PPP). M1 macrophages have increased iNOS levels and secrete various pro-inflammatory cytokines like TNF-α. During normal homeostasis, adipose tissue is enriched with alternatively activated macrophages (M2) that rely mostly on oxidative phosphorylation for its energy requirements. Increased PPARγ and PGC1-β regulates lipid metabolism with increased fatty acid oxidation (FAO). mTOR also plays a crucial role in modulating lipid metabolism and oxidative phosphorylation. M2 macrophages are characterized by Arg-1 expression and anti-inflammatory cytokines like IL-10.

Macrophages make up the largest subset of adipose-infiltrating immune cells and play an important role in maintaining adipose tissue homeostasis. Macrophages are a type of phagocytic cell responsible for clearing cellular debris, pathogens, foreign particles, malignant cells, and other particulates. There are two main phenotypes of activated macrophages, M1 and M2, that are either pro-inflammatory or promote metabolic homeostasis, respectively (Figure 4) [85]. Within adipose tissue, there is a unique population of resident macrophages known as adipose tissue macrophages (ATMs). ATMs in lean adipose tissue generally display an M2-phenotype, expressing genes such as arginase 1 and IL-10. Obesity induces a phenotypic switch of macrophages in the adipose tissue toward a pro-inflammatory M1 state [86]. These activated macrophages then secrete pro-inflammatory cytokines such as TNF-α and induce inflammatory signaling within the adipose microenvironment [87].

Cytokines produced within the adipose tissue are also capable of modulating the phenotype of macrophages. This functional modulation is directly tied to the metabolism of macrophages. For example, in the adipose microenvironment, eosinophils are a major source of IL-4 and are responsible for the maintenance of M2 macrophage populations within adipose tissue [88]. Other studies have shown that adipocytes themselves produce IL-4 and another Th2 cytokine, IL-13, which induces upregulation of lipid metabolism in M2 alternatively activated macrophages [89,90]. The induction of lipid metabolism in M2 macrophages is mediated by IL-4 activation of PPARγ/δ. Myeloid-specific deletion of PPARγ/δ results in impaired numbers of M2 macrophages and increased diet-induced obesity [89,91]. The M1 phenotype, in contrast, is driven by the activation of glycolysis and a concurrent reduction in oxidative phosphorylation [92,93]. Importantly, several studies demonstrate that the abundance of lipids in the adipose tissue causes the adipose tissue macrophages to utilize FAO as the primary source of energy [25,94,95].

In most contexts, tumor-associated macrophages (TAMs) are pro-tumorigenic evidenced by their association with worse prognosis [96,97]. The mechanisms by which TAMs promote tumor growth and metastases are diverse. Adipose-infiltrating macrophages were shown to secrete pro-angiogenic proteins such as vascular endothelial growth factor (VEGF), TNF-α, granulocyte-macrophage colony-stimulating factor (GM-CSF), IL-1, and IL-6 in a breast tumor mouse model [98]. In ovarian cancer, tumor cells and TAMs produce the chemokine CCL22, which mediates the recruitment of T_reg_ cells to the tumor, suppressing anti-tumor immunity [52]. However, a more recent meta-analysis of the prognostic significance of TAMs in ovarian cancer found that by delineating macrophage phenotype, a higher M1/M2 ratio in ovarian cancer was associated with improved survival [99]. Recent studies have suggested that obesity induces the generation of a pro-inflammatory, metabolically activated, adipose tissue-associated macrophage phenotype that is both mechanistically and functionally distinct from the anti-tumor M1 phenotype [100]. Moreover, these obesity-associated macrophages accumulate in the mammary adipose tissue of humans and mice and are responsible for triple-negative breast cancer tumorigenesis [100]. These studies suggest that targeting metabolically active macrophages in adipose tissue could be a viable strategy to prevent tumorigenesis. More critically, pharmacological targeting of the “metabolic” state of the macrophages in the adipose tissue might provide an opportunity to reprogram TAMs into anti-tumor macrophage phenotype.

### 4.2. Eosinophils

Eosinophils are a relatively rare yet important innate cell type that modulates the immune response through antigen presentation to T cells, suppression of inflammation, and maintenance of metabolic homeostasis [101]. Adipose tissue eosinophils protect against diet-induced obesity and associated metabolic dysregulation through their modulation of macrophage alternative activation [88]. However, significant evidence suggests that within the tumor microenvironment, eosinophils can display both tumor-promoting and tumor-suppressive effects [102]. Eosinophilia, the expansion of eosinophils, in cancer patients was first described many decades ago, but the functional consequences of this phenomenon is not completely clear. Eosinophilia with the tumor microenvironment appears to correlate with improved survival in multiple different cancer types [103]. Many significant studies have established the opposing roles of eosinophils on tumor progression both in vitro and in vivo. For example, it was demonstrated that the adoptive transfer of activated eosinophils leads to abrogation of tumor growth via induction of M1 macrophages, leading to infiltration of CD8 T cells in melanoma models in mice [104]. Moreover, eosinophils can also have a direct anti-tumor effect on the cancer cells by the release of IL-4 in the tumor microenvironment [105]. Notably, it was also demonstrated that eosinophils can prevent tumor metastasis and growth of primary tumors in a IL33 dependent manner [106].

Though fewer direct mechanistic studies exist, the pro-tumorigenic role of eosinophils has also been repeatably documented. Studies have shown that eosinophils can secrete several growth factors that can promote tumor growth, such as EGF, FGF, TGFβ1, PDGF, and VEGF. Analogous to adipose tissue, eosinophil-derived IL-13 is also responsible for driving the M2 immune-suppressive macrophage phenotype [107]. Indoleamine 2,3-dioxygenase (IDO) is a tryptophan-catabolizing enzyme that plays an immune-suppressive role in the tumor microenvironment. Tumor-infiltrating eosinophils in non-small cell lung cancer have been shown to express IDO and the presence of IDO-positive eosinophils correlated with a worse overall prognosis [108]. This evidence strongly suggests that eosinophils play an important role in modulating tumor growth and metastasis through a variety of mechanisms. It is currently unknown if the pleiotropic tumor-associated function of eosinophils is due to diverse phenotypes, as is the case with macrophages.

### 4.3. Neutrophils

Composing 50–70% of total circulating leukocytes, neutrophils are known major effectors of acute inflammation with growing evidence for their contribution to chronic inflammation and adaptive immune responses [109,110]. In lean mice, neutrophils compose less than 1% of total immune cells in adipose tissue [111]. In subcutaneous fat, the abundance of neutrophils was significantly correlated with increased BMI and blood pressure [112]. Neutrophils utilize three primary effector functions for clearing pathogens: phagocytosis, degranulation, and neutrophil extracellular trap (NET) formation or NETosis [113]. Neutrophils are largely glycolytic, but studies have described the utilization of additional pathways including oxidative phosphorylation and fatty acid oxidation [114]. Uniquely produced and released by neutrophils, NETs are web-like scaffolds composed primarily of chromatin with cytotoxic proteins and proteases, which immobilize and kill invading pathogens [115]. However, in the context of cancer, NET formation is largely pro-tumorigenic. In mice and humans, NETs are produced in response to ovarian tumor-derived inflammatory factors and bind to ovarian cancer cells, promoting omental metastasis [116].

In an orthotopic ovarian cancer model, a neutrophil influx into milky spots of the omentum prior to metastasis has been described. Within these milky spots, the frequency of polymorphonuclear myeloid-derived suppressor cells (PMN-MDSCs), a form of pathologically activated neutrophils, contributes negatively to the outcomes of cancer therapies. Additionally, fatty acid transport protein 2 (FATP2) has been shown to be upregulated in mouse and human PMN-MDSCs. FATP2 inhibition in mouse PMN-MDSCs leads to a significant decrease in tumor progression [117]. In a retrospective analysis of an immune checkpoint blockade clinical trial of epithelial ovarian cancer, early discontinuation due to poor treatment prognosis was associated with a high neutrophil-to-lymphocyte ratio [118]. While neutrophils have been investigated as potential novel therapeutic targets due to their contribution to tumor progression [119], potential therapies targeting neutrophil metabolism have been largely overlooked. Considering their highly glycolytic phenotype, therapies that target glucose uptake and utilization may be effective in mitigating neutrophil function within the tumor microenvironment.

### 4.4. Innate Lymphoid Cells (ILCs)

ILCs are divided into several different groups that are defined by their patterns of cytokine production and transcription factor expression. ILC1s, ILC2s, and ILC3s bear a resemblance to the T helper cell subsets Th1, Th2, and Th17 cells, respectively, that were described previously. ILCs are largely tissue-resident and have been found to play expansive roles in tissue homeostasis through the shaping of both the innate and adaptive immune responses [120]. Studies have demonstrated a role for ILCs in the regulation of obesity. ILC1s have been shown to be resident in adipose tissue both in lean and obese states [121]. ILC1s promote inflammation mediated by IFNγ and polarize macrophages towards an M1 phenotype [122]. However, in a lean state, adipose tissue ILC1s were found to display cytotoxic activity towards M1 macrophages, which was impaired in the obese state [121]. More research is needed to fully identify whether ILC1s play a direct pathological role in obesity or are bystanders.

ILC2s have been found to contribute to the maintenance of the lean state. ILC2 populations within adipose tissue are maintained by IL-33 where they regulate infiltration of T_reg_ cells, eosinophils, M2 macrophages, and the browning of WAT to limit obesity [123,124]. Very few studies have investigated the role of ILC3s in obesity. However, ILC3s produce IL22, which has been shown to have a protective effect against metabolic disorders [125,126]. Conversely, in cancer, IL22 produced by colonic ILC2-like cells promotes inflammation and proliferation of colonic epithelial cells, leading to dysplasia and colon cancer formation [127]. In other tumor contexts, ILC2s have been shown to have tumor inhibitory function. For example, in several mouse models, ILC2s were shown to directly induce tumor cell apoptosis through an IL33-CXCR2 ligand axis [128]. Given their tissue-resident characteristics, ILCs may be unique tools that can be used to infiltrate the tumor microenvironment.

### 4.5. NK and iNKT Cells

Natural killer (NK) cells are a unique type of innate lymphocyte that displays features of both innate and adaptive immunity and are enriched in adipose tissue [121,122,129]. NK cell function is tightly regulated through interactions with inhibitory and activating signals expressed on cells throughout the body. The cytolytic ability of NK cells provide a rapid host defense against infection and cancer [130]. Notably, NK cells play a central role in anti-tumor immunosurveillance [131]. The first evidence of the role of NK cells in anti-tumor immunity came from studies in the 1980s, which showed that individuals with genetic deficiencies in NK cell activity, had increased incidence of cancer. Further evidence came from a landmark longitudinal study of the peripheral blood of individuals within the general population, which showed that increased cytotoxic NK cell activity correlated with reduced cancer risk [132]. Deletion of the NK cell stimulatory receptor, NKG2D, results in defective immunosurveillance in transgenic mouse models of spontaneous cancer [133]. Similar to T cells, NK cell activation and effector function are dependent on the engagement of glycolysis [134,135]. However, obesity induces lipid accumulation driven by PPARα and PPARδ, which causes dysfunction of NK cell metabolism, reduced production of IFNγ, and reduced tumor cell killing [136]. These results are particularly important in the context of obesity-associated cancers. Therapies which prevent lipid uptake into NK cells may be particularly relevant to maintain their crucial role in anti-tumor immunosurveillance.

Invariant natural killer T (iNKT) cells are a distinct immune subset that shares some characteristics with NK cells and conventional T cells. However, iNKT cells express a semi-invariant T cell receptor α-chain that is restricted to glycolipids presented on CD1d as opposed to conventional T cells, which are MHC-restricted [131]. iNKT cells can modulate immune responses through cytokine expression, interactions with antigen-presenting cells, and direct cytotoxicity. In human and murine WAT, iNKT cells are specifically enriched while their numbers are depleted in obesity and cancer. Adoptive transfer of iNKT cells into obese mice or in vivo activation of iNKT cells led to a reduction in body fat and enhanced insulin sensitivity [137,138]. Deletion of CD1d on adipocytes in obese mice diminishes the iNKT cell response, which results in amplified adipose tissue inflammation and insulin resistance [139,140]. In cancer, iNKT cells have been shown to play a crucial role in anti-tumor immunity. Mice deficient in iNKT cells were incapable of IL-12-mediated rejection of tumors [141]. iNKT cells were also necessary for the rejection of spontaneously formed tumors in mice [142]. For reasons that are not yet fully understood, iNKT cells under some contexts can suppress the immune response by inducing a Th2 response [143]. Overall, these studies indicate that iNKT cells play a significant role in immune regulation to maintain metabolic homeostasis within adipose tissue and in anti-tumor immunosurveillance.

## 5. Conclusions

Tumor-promoting inflammation, deregulating cellular energetics, and avoiding immune destruction are hallmarks of cancer [144]. Collectively, these studies show that the complex interplay within adipose tissue during obesity can lead to the establishment of a pro-inflammatory niche that favors tumor development. The lipid-rich environment of adipose tissue is exploited by cancer cells to fuel tumorigenesis and metastasis. In obesity, CD8 T cells are activated, leading to enhanced inflammation. However, in tumors, it is clear that lipids can cause dysfunction of NK cells and T cells. Future studies that take advantage of relevant models of obesity combined with tumor development will be necessary to fully understand the signaling cascades that are responsible for this dichotomy. Furthermore, many of the findings discussed here are based on work using mouse models. While mice continue to be an essential model system for both cancer and immunology fields, the VAT deposits in mice are dissimilar from humans. Therefore, a more systematic investigation of human VAT would provide essential insight into adipose tissue crosstalk with the immune system and cancer.

In conclusion, many components of the adipose tissue microenvironment modulate the immune response to obesity and cancer. To develop successful therapies for tumors that thrive in the adipose-rich environment, it is necessary to understand the complex relationships between adipose tissue, immune cells, and cancer cells. In particular, fatty acid metabolism and oxidative phosphorylation play a critical role in the function of T_reg_ cells, memory T cells, and macrophages within adipose tissue. We hope that with a greater investigation of the adipose tissue microenvironment, we may begin to develop novel modulators of immunometabolism, which will promote anti-tumor immunity. Metabolic reprogramming of immune cells through the modulation of lipid uptake and utilization may increase the efficacy of current immunotherapies to improve patient survival.
